# Status and related factors of professional growth among young nursing talents: a cross-sectional study in China

**DOI:** 10.1186/s12912-024-01790-7

**Published:** 2024-02-15

**Authors:** Xiuwen Chen, Liqing Yue, Bingyu Li, Jun Li, Xiuying Wu, Bin Peng, Ziwei Cao

**Affiliations:** 1 Teaching and Research Section of Clinical Nursing, National Clinical Research Center for Geriatric Disorders, Xiangya Hospital, Xiangya Hospital of Central South University, Central South University, Changsha, China; 2 National Clinical Research Center for Geriatric Disorders, Xiangya Hospital, Central South University, Changsha, China; 3 Xiangya School of Nursing, Central South University, Changsha, China

**Keywords:** Young nursing talents, Nurse management, Professional growth, Cross-sectional study

## Abstract

**Background:**

The shortage of nurses has been a global human resources problem. A good professional growth environment is essential to developing potential nursing students and attracting nurses to join, and it has great significance in reducing nurse turnover. However, nurses’ comprehensive perceptions of professional growth have not yet been examined.

**Methods:**

A cluster sampling method was used to conduct a professional growth questionnaire survey on young nursing talents from a large Chinese public tertiary A hospital in March 2022.

**Results:**

The score of professional growth among 243 young nursing talents was 57.92 ± 9.607, with a scoring rate of 77.23%. The scores for dimensions of professional growth, from lowest to highest, were rehabilitation growth, promotion speed, professional goal progress, and professional ability development. Attitudes towards participating in training, service as the quality manager or clinical teacher, self-efficacy, professional title, work-family support, education, and organizational commitment of young nursing talents were significantly associated with professional growth.

**Conclusion:**

The professional growth of young nursing talents was at a moderate level and needed to be strengthened. Nursing leaders and managers are expected to develop management practices to enhance young nursing talents’ professional growth in combination with the related factors.

## Introduction

The shortage of nurses has been a global human resources problem. The World Health Organization (WHO) emphasized in the State of the World’s Nursing Report 2020 that no country in the world had a nursing workforce commensurate with universal health coverage and the Sustainable Development Goals, and the global shortage of nurses was estimated at 5.9 million [1]. As the world’s largest developing country, China is facing a more severe shortage of nurses due to its aging population. According to data from the National Bureau of Statistics in 2020, the proportion of people aged 65 and above in China will increase to 13.5%. China is expected to enter an aging society during the 14th Five-Year Plan period. Yet, there are only 32 registered nurses per 10,000 people [2]. Healthcare institutions are facing an unprecedented shortage of registered nurses [3]. Another concern is that the turnover has further exacerbated the shortage of nurses [4]. More and more evidence showed that improving the professional growth environment of nurses was one of the most effective ways to reduce turnover and solve the shortage of nursing human resources [5, 6].

Professional growth refers to the speed of an employee’s career progress within the organization, including career goal progress, career ability improvement, promotion, and compensation growth rate [7]. A good professional growth environment is essential to developing potential nursing students and attracting nurses to join. And also has great significance in reducing turnover and brain drain and is closely related to the quality and safety of nursing [8]. Yu et al. [9] indicated that dissatisfaction with the current professional growth status, such as limited opportunities for further professional education, low salaries, and limited development space, was the main reason for nurses to resign, especially among young nursing talents. Nowadays, with the context of historic levels of nursing workforce shortage, burnout, resignation, and retirement, some studies are increasingly calling for new personnel deployment models [10, 11]. The core discussion of these studies was to develop strategies for expanding young nursing talents [10, 11].

The departure of outstanding talents within an organization poses a significant risk of substantial losses. Numerous empirical studies underscore the pivotal role of professional growth as a determinant in individuals’ job selection process [12–14]. Young nursing talents are the new generation of nursing managers in the future, the key to the construction of the hospital talent echelon, and important elements to enhance the comprehensive strength of the hospital [15]. Thompson et al. [16] called managers should consider the professional growth of young nursing talents to be closely related to the development of the hospital and pay attention to the professional growth, create more educational and practical opportunities for young nursing talents, and develop priority skills. However, existing research on the nurse training curriculum predominantly concentrates on the training of novice nurses, with limited studies explicitly addressing the unique needs of young nursing talents.

Recognizing the current status of professional growth among young nursing talents and understanding their specific requirements are imperative prerequisites for the formulation of effective talent recruitment and retention strategies. Moreover, this understanding lays the foundation for the development of targeted nurse training curricula. Nowadays, some studies have emphasized the importance of professional growth in the nursing profession, recognizing it as a critical factor for individual career satisfaction and overall healthcare quality [12, 17]. However, a significant gap exists in the current literature concerning the specific needs and challenges faced by young nursing talents in their professional growth journey. Therefore, the purpose of this study is to explore the current situation of the professional growth of young nursing talents and analyze the relevant factors so as to provide a basis for departments to formulate and optimize the training mode of young nursing talents.

## Methods

### Study design

A cross-sectional study was conducted to determine the status and related factors of professional growth among young nursing talents in March 2022.

### Participants

A cluster sampling method was used to conduct a professional growth questionnaire survey on young nursing talents from a large public tertiary A hospital in Hunan Province, China. This hospital is also a teaching hospital and issued a notice on *the training plan for young nursing talents* to select the first batch of young nursing talents in 2022. The selection criteria for nursing talents were as follows: (1) young nurses who work in clinical nursing positions and have not held the position of head nurse; (2) age: no more than 40 years old; (3) education: Full-time undergraduate or above, or those who have obtained a master’s degree in nursing and related fields; (4) nursing work experience: at least 3 years of experience in this hospital; (5) title: nurse practitioner; (6) annual assessment: passed or above the annual assessment in the past three years; (7) those who meet the following conditions were preferred: having a master’s or doctoral degree; or having served as the head teacher of a department; or having led research projects at the hospital or above level. The setting of the number of young nursing talent selection was: if the number of nurses in the nursing unit were less than 60, 1 person would be selected, and if the number of nurses in the nursing unit was 60 or more, a ratio of 60:1 would be applied (rounded). The selection process for young nursing talents consisted of four steps: individual self-registration, democratic recommendation and selection organized by each nursing unit, review by the nursing department, and resolution by the hospital party committee. All the selected young nursing talents were participants in this study.

### Sample size and setting

Considering that the regression analysis method was selected for this study, the sample size was recommended to be at least 10 times the number of explanatory variables [18]. It was estimated that there were 15 explanatory variables in this study, and 20% of invalid questionnaires were also considered. Therefore, a minimum of 180 young nursing talents was required.

### Instrument

The instrument mainly consisted of five sections: the demographics and work characteristics questionnaire, the Professional Growth Scale, the General Self-Efficacy Scale, the Work-family Support Scale, and the Organizational Commitment Questionnaire.

#### Demographics and work characteristics

Demographics and work characteristics were designed by the researcher, including age, gender, years of work, education, title, position, career development direction, attitudes toward training, etc.

#### Professional growth scale

The Professional Growth Scale was originally developed in Chinese by Weng [19] in 2009. It consisted of 15 items with four dimensions: professional goal progress (items 1 ~ 4), professional ability development (items 5 ~ 8), promotion speed (items 9 ~ 12), and remuneration growth (items 13 ~ 15). All options of this scale were scored on a positive five-point Likert scale, with scores ranging from 1 to 5 for “very inconsistent” to “very consistent.” The total score on the scale was 75 points, and the higher the score, the better the professional growth situation. The Cronbach’s alpha was 0.943, and the Cronbach’s alpha coefficient for each dimension ranged from 0.890 to 0.929, indicating acceptable reliability.

#### General self-efficacy scale

The General Self-Efficacy Scale, developed by Schwarzer, was translated and adapted into Chinese [20]. It consists of 10 items and mainly measures an individual’s evaluation of their problem-solving abilities. All options of this scale were scored on a positive four-point Likert scale, with scores ranging from 1 to 4 for “completely incorrect” to “completely correct.” The total score on the scale was 40 points, and the higher the score, the better the sense of efficacy. The Cronbach’s alpha coefficient of the questionnaire was 0.91, indicating the acceptable reliability of the scale.

#### Work-family support scale

The Work-family Support Scale was originally developed in Chinese by Li et al. [21], with a total of 30 items. The scale was divided into two parts: work support and family support. Likert 5-level scoring method was adopted, ranging from “totally inconsistent” to “completely consistent,” and the higher the score was, the more work-family support nurses received. Cronbach’s alpha coefficient of the entire scale was 0.969.

#### Organizational commitment questionnaire

The Organizational Commitment Questionnaire was originally developed in Chinese by Ling et al. [22], with a total of 25 items, to measure the emotional questionnaire of employees who fully participate in the work of the organization. There are 5 dimensions of emotional commitment: normative commitment, ideal commitment, economic commitment, and opportunity commitment. All options of this questionnaire were scored on a positive five-point Likert scale, with scores ranging from 1 to 5 for “strongly disagree” to “strongly agree.” The higher the score, the better the organizational commitment of nurses. The Cronbach’s alpha coefficient of the entire questionnaire was 0.919.

### Data collection and ethical considerations

Ethical approval was obtained by the Ethics Committee of Xiangya Hospital of Central South University (No: 202,103,460). Before the study began, all the participants were informed about the purpose and process of this study. The Declaration of Helsinki guidelines were followed (World Medical Association, 2013). Data were collected in March 2022 through an electronic questionnaire in the form of a Questionnaire Star. The questionnaire was filled out independently by the participants voluntarily. In order to ensure the quality of the electronic questionnaire, questionnaires were collected at the first class of the training course for young nursing talents. The questionnaire used a unified guide to explain the concept of professional growth, the meaning and the way to answer. In addition, in order to avoid repeated filling in the questionnaire, this study set the same mobile phone device on the survey platform to fill in only once. The researchers recovered the survey results directly through the background of the official website of Questionnaire Star. The questionnaire was numbered automatically according to the order of completion of the questionnaire, and the contents of the questionnaire were checked and deleted by two persons. A total of 248 questionnaires were recovered in this study. Since the pre-experimental test required at least 2 min to answer questions, a total of 5 questionnaires with less than 2 min were excluded, and 243 valid questionnaires were finally recovered, with an effective recovery rate of 97.98%.

### Statistics methods

SPSS 22.0 software was used for data processing and analysis. Counting data such as gender and title were described by frequency and component ratio, and two independent samples *T*-test or *one-way ANOVA* were used to study whether there were statistical differences in nurses’ professional growth among different groups. After the normality test, the data were normally distributed, so the measurement data, such as age and working years, were described by means and standard deviations (*SD*), and the correlation between variables was tested by *Pearson* correlation analysis. Multiple linear regression analysis was used for related factors, and *p* < 0.05 was considered statistically significant. In addition, the score rates for the overall questionnaire and each dimension were calculated as follows: actual scores / theoretical maximum × 100%.

## Results

### Sociodemographic characteristics of the sample

243 young nursing talents were finally included, of whom 8 (3.3%) were male, and 235 (96.7%) were female. The age of young nursing talents concentrated at 30 ~ 35 years old, accounting for 65.8%. The title of young talented nurses was mainly in charge of nurses, accounting for 83.1%. Education distribution was mainly bachelor’s degrees, accounting for 70.0%, and master’s degrees accounted for 30.0%. The demographic characteristics of the participants and their correlations with professional growth are shown in Table 1. The results showed that there were significant differences in professional growth scores among young nursing talents with different educational professional titles, service as the quality manager or clinical teacher, and attitudes toward participating in training (*P* < 0.05).

**Table 1:** Sociodemographic characteristics correlation with young nursing talents’ professional growth (*N* = 243)

Variable	Frequency (%)	Total scores of the questionnaire	
		Mean ± SD	Test results
**Gender**			
Female	235 (96.7)	57.74 ± 9.527	*t* = 0.241
Male	8 (3.3)	63.25 ± 11.081	*P* = 0.111
**Age (years)**			
≤ 25	5 (2.1)	61.40 ± 9.370	*F* = 1.306
26 to 30	43 (17.7)	59.33 ± 9.778	*P* = 0.273
31 to 35	160 (65.8)	57.07 ± 10.080	
36 to 40	35 (14.4)	59.60 ± 6.536	
**Education**			
Bachelor degree	170 (70.0)	57.02 ± 9.643	*t*=-2.258
Master degree	73 (30.0)	60.03 ± 9.487	*P* = 0.025
**Professional title**			
Primary	39 (16.1)	60.05 ± 10.221	*F* = 3.509
Intermediate	202 (83.1)	57.37 ± 9.392	*P* = 0.031
Senior	2 (0.8)	72.00 ± 0.000	
**Years of experience**			
≤ 5	16 (6.6)	60.44 ± 10.551	*F* = 1.317
6 to 10	99 (40.7)	57.65 ± 9.776	*P* = 0.264
11 to 15	114 (46.9)	57.36 ± 9.456	
16 to 20	9 (3.7)	59.11 ± 7.390	
>20	5 (2.1)	66.00 ± 8.246	
**Service as the quality manager or clinical teacher**			
Yes	145 (59.7)	59.78 ± 8.892	*t* = 2.500
No	98 (40.3)	56.67 ± 9.895	*P* = 0.013
**Employment form**			
Authorized personnel	197 (81.1)	57.62 ± 9.805	*F* = 0.638
Contract system	41 (16.9)	59.46 ± 8.975	*P* = 0.529
Others	5 (2.0)	57.20 ± 6.140	
**Marital status**			
Unmarried	50 (20.6)	59.14 ± 9.731	*F* = 1.106
Married	191 (78.6)	57.53 ± 9.539	*P* = 0.333
Others	2 (0.8)	65.00 ± 14.142	
**Are you the only child of your parents**			
Yes	73 (30.0)	57.78 ± 9.505	*t*=-0.150
No	170 (70.0)	57.98 ± 9.677	*P* = 0.881
**Are you a specialist nurse**			
Yes	133 (54.7)	57.97 ± 8.719	*t* = 0.086
No	110 (45.3)	57.86 ± 10.622	*P* = 0.932
**Attitudes towards participating in training**			
Like	235 (96.7)	58.29 ± 9.251	*t* = 3.298
Dislike	8 (3.3)	47.13 ± 13.861	*P* = 0.001
**Preferred career direction**			
Nursing management	93 (38.3)	59.31 ± 8.502	*F* = 2.546
Nursing specialty	136 (56.0)	57.40 ± 9.649	*P* = 0.081
Others	14 (5.7)	53.71 ± 14.280	

Note: *P <0.05

### Scores of professional growth

The results of this study showed that the total score on the Professional Growth Scale among young nursing talents was 57.92 ± 9.607, with a score rate of 77.23%. The mean scores of each dimension from lowest to highest were remuneration growth (2.38 ± 0.757), promotion speed (3.70 ± 0.828), professional goal progress (4.11 ± 0.694), and professional ability development (4.29 ± 0.580), with a scoring rate of 63.53%, 73.95%, 82.20%, and 85.80%, respectively. The score of each item is shown in Table 2.

**Table 2:** The score of each item of the Professional Growth Scale among young nursing talents(*n* = 243)

Items	Scores (Mean ± SD)
1. My current job brings me closer to my career goals	4.02 ± 0.784
2. My current job is related to my career goal and ideal	4.11 ± 0.758
3. My current job has laid the foundation for my career goals	4.26 ± 0.694
4. My present job offers me a good opportunity for development	4.06 ± 0.816
5. My current job enables me to acquire new skills related to my job	4.24 ± 0.675
6. My current job can help me acquire new knowledge related to my job	4.28 ± 0.621
7. My current job can help me accumulate richer experience	4.37 ± 0.591
8. My current job has trained and improved my professional ability	4.26 ± 0.665
9. In my current workplace, my position has improved rapidly	4.05 ± 0.789
10. In my current workplace, there is a high possibility of my position being promoted	3.63 ± 0.951
11. In my current work unit, my position is more ideal than another unit	3.70 ± 1.027
12. Compared with my colleagues, I was promoted more quickly	3.42 ± 1.031
13. My salary is rising faster at my current workplace	3.23 ± 1.061
14. My salary may be increased at my current workplace	3.33 ± 1.012
15. Compared with my colleagues, my salary has gone up faster	2.98 ± 1.153

### Correlation analysis of general self-efficacy, work-family support, organizational commitment, and professional growth among young nursing talents

The results showed that the total scores of the General Self-Efficacy Scale, Work-family Support Scale, and Organizational Commitment Questionnaire were 30.22 ± 6.191, 122.61 ± 18.431, and 88.59 ± 19.773, with scoring rates 75.55%, 81.33% and 70.87% respectively. Pearson correlation analysis showed that self-efficacy, work-family support, and organizational commitment scores were positively correlated with professional growth scores, as shown in Table 3.

**Table 3:** Correlation analysis of general self-efficacy, work-family support, organizational commitment, and professional growth among young nursing talents

	Professional growth scores	General self-efficacy scores	Work-family support scores	Organizational commitment scores
Professional growth scores	1			
General self-efficacy scores	0.703**	1		
Work-family support scores	0.733**	0.595**	1	
Organizational commitment scores	0.681**	0.622**	0.634**	1

Note: **P <0.01

### Multiple Linear regression analysis on the professional growth of young nursing talents

Multiple linear regression analysis was performed. The overall scores of the professional growth were included as dependent variables, and the education, professional title, service as the quality manager or clinical teacher, attitudes towards participating in training, general self-efficacy, work-family support, and organizational commitment were included as independent variables. Variance analysis of the regression equation showed that the *F* value was 70.535 (*P* = 0.001), indicating that the fitted multiple linear regression equation was statistically significant. The complex correlation coefficient *R* = 0.678 and determination coefficient *R*^2^ = 0.823 indicated that these seven variables explain 67.8% of the variation. In addition, the standard regression coefficient showed that the influencing factors in descending order were attitudes towards participating in training, service as the quality manager or clinical teacher, self-efficacy, professional title, work-family support, education, and organizational commitment, as shown in Table 4.

**Table 4:** Results of multiple linear regression analysis

Variable	B-Value	Standard Error	***t***	***P***
Constant	11.975	4.659	2.570	0.011
Attitudes towards participating in training	-1.526	2.082	-0.733	0.464
Service as the quality manager or clinical teacher	-1.056	0.773	-1.365	0.173
General self-efficacy	0.485	0.080	6.056	0.000
Professional title	-0.451	0.985	-0.458	0.647
Work-family Support	0.199	0.027	7.471	0.000
Education	0.118	0.801	0.147	0.883
Organizational Commitment	0.115	0.025	4.506	0.000

## Discussion

### The professional growth of young nursing talents

This study showed that the scoring rate of professional growth among young Chinese nursing talents was 77.23%. Generally, under a percentage marking system, it is ideal when the score is above 80 in China [2]. The participants included in this study were young nursing talents who were outstanding among all nurses, but their professional growth score rate was still lower than 80%. It can be seen that there is still much room for improvement in the professional growth level of young nursing talents in China. A number of studies have shown that improving the professional growth of nurses was not only conducive to reducing the loss of nursing talents but also reduced the burnout of nurses, promoted enthusiasm for work, and produced more creative results [23, 24]. Therefore, nursing managers should pay attention to the professional growth of young nurses, broaden the career development path, and actively explore the talent training model. The results of this study also unveiled that the dimension in which young nursing talents received the lowest was remuneration growth. It was consistent with the previous studies [5, 25]. Lai et al. [25] carried out a survey on the status of the nursing practice environment among Chinese nurses, and the results also showed that nurses were dissatisfied with the remuneration package. However, Kim et al. [26] pointed out that salary was the most important decision factor for nurse turnover. Therefore, the managers of China’s third-level A hospitals still need more exploration in terms of the growth of organizational returns, and the establishment of a scientific and reasonable salary distribution system has become an urgent management problem. Fortunately, the results of this study showed that the scores in the dimension of professional ability development were satisfactory, with a scoring rate of 85.80%, and the entry with the highest score, “*My current job can help me acquire new knowledge related to my job”* also belongs to this dimension. Young nursing talents recognized that nursing work could bring improvement in skills and knowledge, which to some extent confirms tertiary hospitals in China have attached great importance to strengthening the training of nurses and improving nurses’ professional ability. In addition, the recognition of professional abilities by young nursing talents may also be due to the outbreak of COVID-19, which further demonstrated the value of the nursing profession to the healthcare system and humanity. In light of these observations, we plan to use the study results as a foundation and reference to launch a systematically designed training program for young nursing talents.

### Correlation analysis of general self-efficacy, work-family support, organizational commitment, and professional growth

The results of this study showed that general self-efficacy, work-family support, and organizational commitment were all positively correlated with professional growth, and the results of multiple regression analysis also demonstrated that these were the main influencing factors for professional growth. Managers can promote the professional growth of young nursing talents by exploring improvement strategies targeting these three factors.

#### General self-efficacy

General self-efficacy refers to the judgment of how well one can execute courses of action required to deal with prospective situations, and individuals with high self-efficacy set their own goals. In contrast, those with low self-efficacy may produce poor outcomes [27]. This study showed general self-efficacy was positively correlated with the professional growth of young nursing talents, which is consistent with previous studies [28, 29]. Ge et al. [29] have proven that individuals with a higher level of self-efficacy could achieve a higher level of professional growth. Research [30] also maintained individuals with high levels of self-efficacy tend to set high-level career goals for themselves, put in more effort, and be more strategic in their pursuit of career success in order to achieve their self-set career goals. Therefore, managers are suggested to improve the professional growth of young nursing talents by enhancing their self-efficacy, such as praising nurses, appreciating nurses, supporting nurses, and caring for nurses in their work.

#### Work-family support

Work-family support is defined as the various supports that employees receive from the organizational and family fields in the course of their work to facilitate the balance between work and family [31]. The results of this study showed that work-family support was positively correlated with professional growth, which was similar to the findings of Yang et al. [32]. The female group has always been the prominent feature of the nursing team, and most nurses need to take care of more family responsibilities while taking care of their work. This may be the reason why family support has a greater impact on the professional growth of young nursing talents. Therefore, this study suggests that managers provide a variety of supportive policies and care for nurses so that employees can deal with family affairs and make more efforts for follow-up work so as to achieve mutual benefit and a win-win situation for employees and enterprises.

#### Organizational commitment

Organizational commitment means the feeling of being willing to fully participate in the work of the organization as the investment in the organization increases [33]. This study revealed that organizational commitment was the main factor affecting the professional growth of young nursing talents. The reason may be that nurses with high organizational commitment give more and invest more and, therefore, have easier access to professional growth opportunities. Berberoglu et al. [34] similarly found that organizational climate can effectively predict 20% of organizational commitment. If employees can be promoted to perceive organizational climate positively, they will have a higher level of organizational commitment. Tadesse et al. [35] revealed nurses with low organizational commitment were found to be 3.7 times more likely to leave the nursing profession. This likelihood of departure was significantly higher compared to nurses with high professional commitment. Therefore, managers are expected to create a good career development and working environment, increase employees’ emotional investment in the organization, or encourage nurses to actively participate in organizational affairs through transformational leadership and structural empowerment. This can encourage employees to generate more identification and thus generate more career commitment.

### Factors associated with professional growth

The results of regression analysis showed that in addition to self-efficacy, work-family support, and organizational commitment, the professional growth of young nursing talents was also related to their education, professional title, service as the quality manager or clinical teacher, and attitudes towards participating in training. The professional growth score of young nursing talents with master’s degrees was higher than that of undergraduate nurses. Lai et al. [25] similarly showed that the higher the education of nurses, the better the professionalism, logic, and learning ability in the working environment, and the better the professional environment. A survey conducted by Cleary and colleagues [36] among undergraduate nursing graduates in Singapore revealed that the graduates believed further education was essential for their professional growth. Limited access to additional education was identified as a significant obstacle to professional development. This finding aligns with the study by Tanaka et al. [37]. In addition, Chen et al. [2] pointed out that nurses with higher education have more development opportunities, and their work ability and management abilities are relatively easier to recognize. Nowadays, tertiary hospitals attach great importance to scientific research work. Nurses with high education tend to have better scientific research thinking and research ability and can get more development opportunities in hospitals. Therefore, young nurses are encouraged to pursue continuing education. It is recommended that hospital administrators consider providing access to further education based on merit rather than relying solely on bureaucratic criteria or hospital sponsorship. Additionally, offering more tailored courses relevant to specific clinical specialties.

At the same time, the results of this study showed that the higher the professional title of nurses, the higher the professional growth score, consistent with previous studies [2, 35]. Within the context of career development, attaining specific professional titles emerges as a noteworthy achievement. These titles go beyond mere honors; they serve as recognition of an individual’s specialized knowledge and skills within a designated field. Such titles encompass professional certifications, qualifications, or industry-specific designations, showcasing an individual’s competence and potentially unlocking diverse career opportunities and elevated remuneration levels. Therefore, when carrying out the training of young nursing talents, managers can set up career planning and management courses, so that young talented nurses can master the evaluation rules of professional title promotion in advance and determine the future career planning scheme. In addition, service as the quality manager or clinical teacher was related to the professional growth of young nursing talents. This suggests that managers can set up more diverse positions, promote the active participation of young nursing talents in organizational affairs, and enhance their enthusiasm.

What’s more, this study displayed that attitudes toward the nursing profession were associated with professional growth. Young nursing talent who liked the nursing profession received higher scores compared to those who disliked it. Ni et al. [6] pointed out that nurses’ identification with their profession is a specific sign of career growth and development, and the stronger nurses’ professional identification, the more likely they are to show higher professional motivation, which may have a positive impact on their professional growth. Meanwhile, AbuAlRub et al. [38] revealed that negative nursing career attitudes can lead to nurses being more inclined to consider leaving their current jobs, reducing career drive and motivation, which is not conducive to career growth and development. It can be seen that enhancing the sense of value of the nursing profession and making young nurses truly love nursing work is very important for the professional growth of young nursing talents. When young nurses have a deep understanding of the value and importance of their profession, they are more likely to maintain a positive professional attitude, seek continuous learning opportunities, and pursue career development. This, in turn, contributes to their overall growth and success in the nursing field.

### Limitations

This study has several limitations that could affect the reliability of the results. Firstly, this study only investigated young nursing talents in a tertiary A hospital, which was not a national survey, and the representative sample may be poor. Secondly, in the analysis of the associations of professional growth, only the relationship between information on the general population and professional growth was analyzed, and this study type could not demonstrate the cause-and-effect relationship. Thirdly, this study adopted the form of an online questionnaire to carry out the survey. Although some questionnaires were excluded according to the answering time, the bias of respondents may be unavoidable. It is suggested that the data collection form should be further improved in future studies to obtain more rigorous and precise conclusions.

### Implication for nursing

Young nursing talents are the new generation of nursing management talents in the future, and are the mainstay of sustainable development of hospitals. A good professional growth environment is of great significance in addressing the shortage of nurses. This study showed that even young nursing talents in tertiary A hospitals still have moderate professional growth scores, indicating that managers need to provide protocols, guidance, courses, training, and support to the young nursing talents. Nursing managers are recommended to regularly evaluate and improve the professional growth level of young nursing talents. In addition, this study also showed that the professional growth of young nursing talents was associated with attitudes towards participating in training, service as the quality manager or clinical teacher, self-efficacy, professional title, work-family support, education, and organizational commitment. It can be seen that medical institutions can improve the professional growth of young nursing talents through the following aspects: strengthen the professional identity education and publicize nursing professionalism to enhance the positive attitudes toward the nursing profession; Develop policies to encourage and support the continuing education of young nursing talents; Provide more opportunities for young nursing talents to participate in hospital management; To create a friendly organizational atmosphere to help young nursing talents improve self-efficacy, family-work support and organizational commitment, so as to create a better professional growth environment.

## Conclusion

This study used a cross-sectional survey method to analyze the professional growth status and related factors of young nursing talents in tertiary A hospitals. The results indicated that the professional growth of young nursing talents was at a moderate level, and the attitudes towards participating in training, service as the quality manager or clinical teacher, self-efficacy, professional title, work-family support, education, and organizational commitment were associated with professional growth among young nursing talents. Nursing leaders and managers are recommended to pay close attention to the professional growth of young nursing talents. They are expected to develop relevant management practices to enhance their professional growth.

## Data Availability

All data generated or analyzed during this study are included in this published article.
